# A synthetic cohort analysis of postoperative management of primary cardiac angiosarcoma and a case report

**DOI:** 10.3389/fonc.2025.1625049

**Published:** 2025-09-15

**Authors:** Ying Cai, Hang Yang, Dan Yuan, Su-Han Jin, Junzhu Xu, Wei Hu, Yuju Bai, Xinjuan Li, Zejin Wang, Dengshen Zhang, Ke Guo, Shixiang Wang, Udo S. Gaipl, Yuan Liu, Hu Ma, Jian-Guo Zhou

**Affiliations:** ^1^ Department of Oncology, The Second Affiliated Hospital of Zunyi Medical University, Zunyi, China; ^2^ Department of Pathology, Affiliated Hospital of Zunyi Medical University, Zunyi, China; ^3^ Department of Orthodontics, Affiliated Stomatological Hospital of Zunyi Medical University, Zunyi, China; ^4^ Department of Imaging, The Second Affiliated Hospital of Zunyi Medical University, Zunyi, China; ^5^ Department of Ultrasound, The Second Affiliated Hospital of Zunyi Medical University, Zunyi, China; ^6^ Department of Cardiac Macrovascular Surgery, Affiliated Hospital of Zunyi Medical University, Zunyi, China; ^7^ Department of Biomedical Informatics, School of Life Sciences, Central South University, Changsha, China; ^8^ Translational Radiobiology, Department of Radiation Oncology, Universitätsklinikum Erlangen, Friedrich-Alexander-Universität Erlangen-Nürnberg, Erlangen, Germany; ^9^ Department of Clinical Pharmacy, Liaocheng Cancer Hospital, Liaocheng, China

**Keywords:** heart tumor, primary cardiac angiosarcoma, radiotherapy, adjuvant chemotherapy, prognosis

## Abstract

**Background:**

Primary cardiac angiosarcoma is a rare and aggressive malignancy originating from the endothelial lining of cardiac blood vessels. The prognosis remains extremely poor. The study was to evaluate postoperative survival in patients with primary cardiac angiosarcoma after treated with adjuvant therapy.

**Methods:**

A systematic review of PubMed from January 1985 to December 2023 was performed to establish a synthetic cohort of patients undergoing surgery for primary cardiac angiosarcoma. Survival analysis was used to assess the relationship between postoperative adjuvant therapy and prognosis. Univariable and multivariable cox regression analyses were used to identify prognostic factors. We then established and validated a nomogram by receiver operating characteristic (ROC) curves, calibration curves and decision curve analysis (DCA). Moreover, we present a case of 49-year-old patient with primary cardiac angiosarcoma.

**Results:**

In the synthetic cohort, the patients with postoperative adjuvant therapy reached longer overall survival (OS) and progression-free survival (PFS) than those without postoperative adjuvant therapy (median OS: 14 VS 8 months, HR = 5.62, 95%CI: 1.66-19.08, P<0.001; median PFS: 12 VS 6 months, HR = 2.98, 95%CI: 1.03-8.66, P = 0.007; Log rank test). Radiotherapy (HR = 0.14, 95% CI: 0.04-0.54, P = 0.004) and chemotherapy (HR = 0.03, 95% CI: 0.00-0.27, P = 0.002) were significantly correlated with better OS. DCA and ROC curves confirmed the nomogram can predict postoperative 6-month survival in patients with primary cardiac angiosarcoma. OS was indistinguishable between patients with R0 or R1 resection (10 VS 10 months, HR = 0.99; 95%CI: 0.34-2.86; P = 0.986). However, compared to patients underwent R1 resection, patients undergoing R0 resection have longer but not statistically significant PFS (10 VS 7 months, HR = 2.16; 95%CI: 0.83-5.61; P = 0.090).

**Conclusion:**

The prognosis of patients with primary cardiac angiosarcoma remains extremely poor, even with surgical resection. Postoperative adjuvant therapy was associated with significantly better survival in a small cohort of patients with primary cardiac angiosarcoma. Further studies are warranted to guide future recommendations.

**Systematic Review Registration:**

https://www.crd.york.ac.uk/prospero/, identifier CRD420251139779.

## Introduction

Primary cardiac angiosarcoma is a rare and highly malignant cardiac tumor, originating from the endothelial lining of cardiac blood vessels and accounting for approximately 25%-30% of all primary cardiac malignancies (1). It is considered to be the most fatal and aggressive primary cardiac malignancy (2). Multicenter studies revealed that primary cardiac malignancies are predominantly sarcomas, followed by lymphomas and mesotheliomas. In sarcomas, angiosarcoma is the most common histological type, while smooth muscle sarcomas and rhabdomyosarcomas are less frequently encountered (3). Cardiac angiosarcoma often affect young males, with a predilection for the right heart, especially right atrium (4, 5). Cardiac angiosarcomas involving the atria are exceedingly scarce. The prognosis of patients with primary cardiac angiosarcoma is considerably poor, with a median overall survival of 6 months (6). The optimal management strategy is still argued, due to the rarity of the tumor (7). Current treatment options include surgery, radiotherapy, and chemotherapy. Surgical resection is an effective treatment of primary malignant cardiac tumors. Patients with cardiac sarcoma who underwent surgery had a median survival of 12 months, compared to only 1 month for those who did not (6). Despite the considered benefits of postoperative adjuvant therapy, clear guidelines or treatment recommendations for radiotherapy and chemotherapy in cardiac sarcoma are currently lacking (8). Further investigation is warranted regarding the postoperative management strategy and prognosis of primary cardiac angiosarcoma patients.

This study included 21 cases of postoperative primary cardiac angiosarcoma in PubMed from January 1985 to December 2023 to establish a synthetic cohort (n=21). Subsequently, univariable and multivariable cox proportional hazards regression analyses were performed to identify prognostic risk factors of primary cardiac angiosarcoma, which included sex, age, race, tumor location, postoperative radiotherapy, and chemotherapy. This was used to establish and validate a postoperative risk prediction model. Simultaneously, we present a case of a 49-year-old patient with primary cardiac angiosarcoma from The Affiliated Hospital of Zunyi Medical University, who underwent partial tumor resection and six cycles of postoperative adjuvant chemotherapy. Based on case report and analysis of the synthetic cohort, this study aims to provide further evidence for establishing effective postoperative management strategies for cardiac sarcoma.

## Case report

A 49-year-old man was admitted to the local hospital for cough, sputum production, exertional dyspnea, and bilateral lower extremity edema after exposure to cold in the past three weeks. After two weeks of anti-infective therapy (levofloxacin, cephalosporins, and symptomatic treatment with antitussive medications), the symptoms showed no improvement. Then the patients presented to the Respiratory Department of the Affiliated Hospital of Zunyi Medical University for further treatment. Upon evaluation, the physical examination revealed a normal cardiac silhouette, a heart rate of 78 beats per minute (bpm) with regular rhythm, and no cardiac murmurs noted on auscultation.

**Figure 1:**
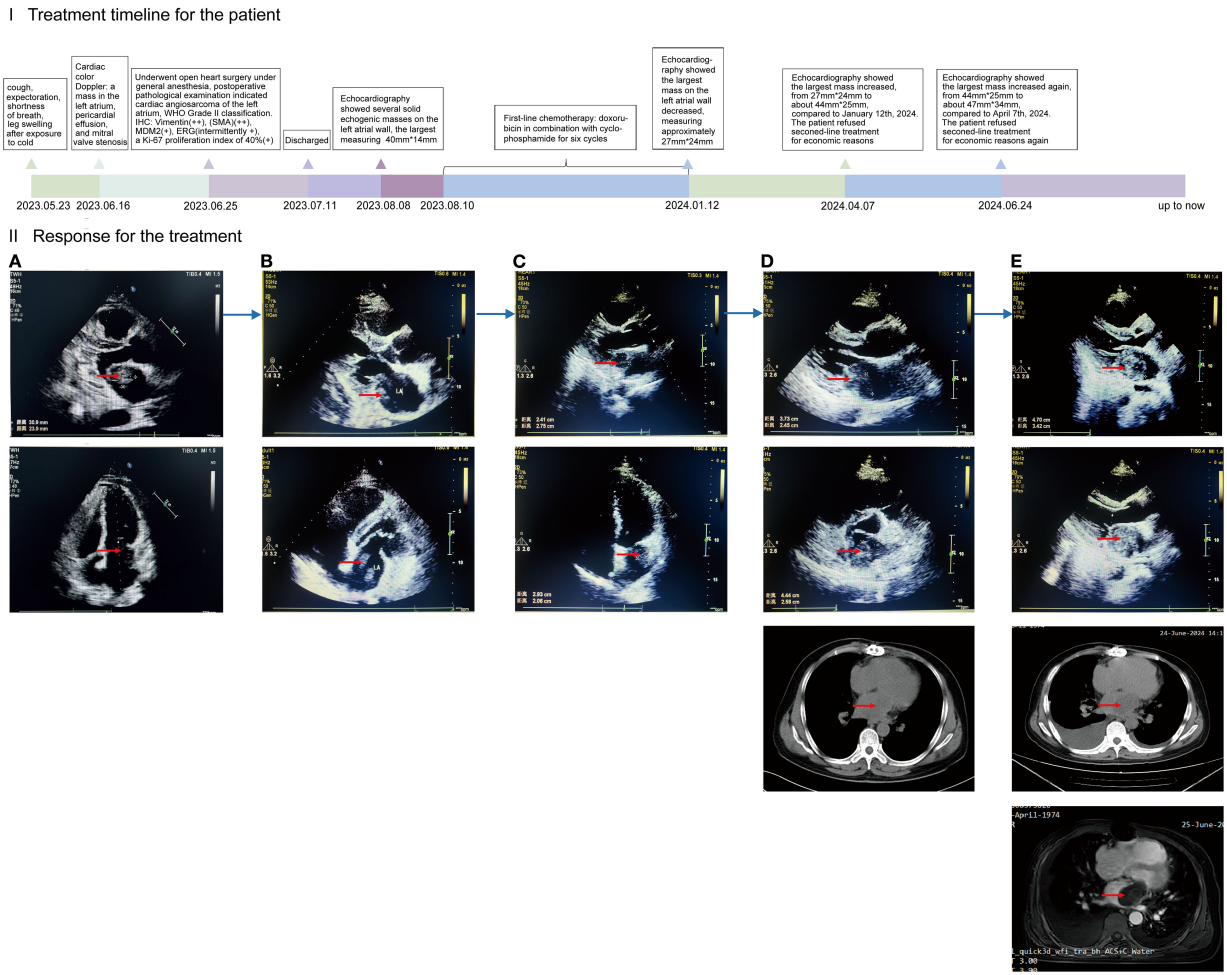
**(I)** (x-coordinate is the time, the unit is months): timeline diagram of the patient with primary cardiac angiosarcoma of disease progression and treatment; the PFS during this treatment period is expressed in length. **(II)**: Efficacy of treatment as demonstrated by cardiac color Doppler ultrasound, chest CT, and cardiac MRI. CT: computed tomography. MRI: magnetic resonance imaging. **(A)** Primary lesions in the heart. Preoperative color Doppler ultrasound, performed ten days prior to surgery, revealed several hypoechoic masses in the left atrium, the largest measuring approximately 30mm*23mm. **(B)** Forty-three days after surgery, cardiac color Doppler ultrasound revealed several irregular solid echogenic masses on the left atrial wall, the largest measuring approximately 40mm*14mm. **(C)** After treatment with six cycles of chemotherapy, the largest mass on the left atrial wall decreased, measuring approximately 27mm*24mm at six and a half months postoperatively. **(D)** Disease progression. The largest mass on the left atrial wall increased, measuring approximately 44mm*25mm at nine and a half months postoperatively. **(E)** Disease progression again. Twelve months postoperatively, the largest mass on the left atrial wall increased, measuring approximately 47mm*34mm.

In addition to left ventricular systolic function assessment, Cardiac color Doppler ultrasound exhibited the following findings (**Figure 1. II-A**).

1. A hypoechogenic mass measuring approximately 30 millimeters x23 millimeters (mm) was attached to the base of the atrial appendage in the left atrium and demonstrated dynamic mobility synchronized with the cardiac cycle, causing diastolic obstruction of the mitral valve orifice.
2. Hypoechoic areas measuring approximately 4 mm and 5mm in thickness were observed in the anterior pericardial sac and beneath the cardiac apex, respectively.
3. Mitral valve obstructive stenosis with a forward flow velocity of approximately 311 centimeters per second (cm/s) and a peak pressure gradient of about 39 millimeters of mercury (mmHg). Mild tricuspid regurgitation was present, and the estimated pulmonary artery systolic pressure, based on tricuspid regurgitation, was approximately 61 mmHg.

After a thorough evaluation and exclusion of surgical contraindications, the patient underwent open heart surgery under general anesthesia to perform partial cardiac tumor resection using extracorporeal circulation. Intraoperatively, numerous varied-sized nodules were observed on the left atrial wall and posterior leaflet of the mitral valve, the largest measuring approximately 10mmx5 mm, alongside a 40x30mm solid mass attached to the left atrial appendage following the incision of the right atrium and interatrial septum. Due to extensive tumor invasion of the left atrial wall and mitral valve, complete resection was not feasible, and the procedure was concluded after partial tumor removal.

**Figure 2:**
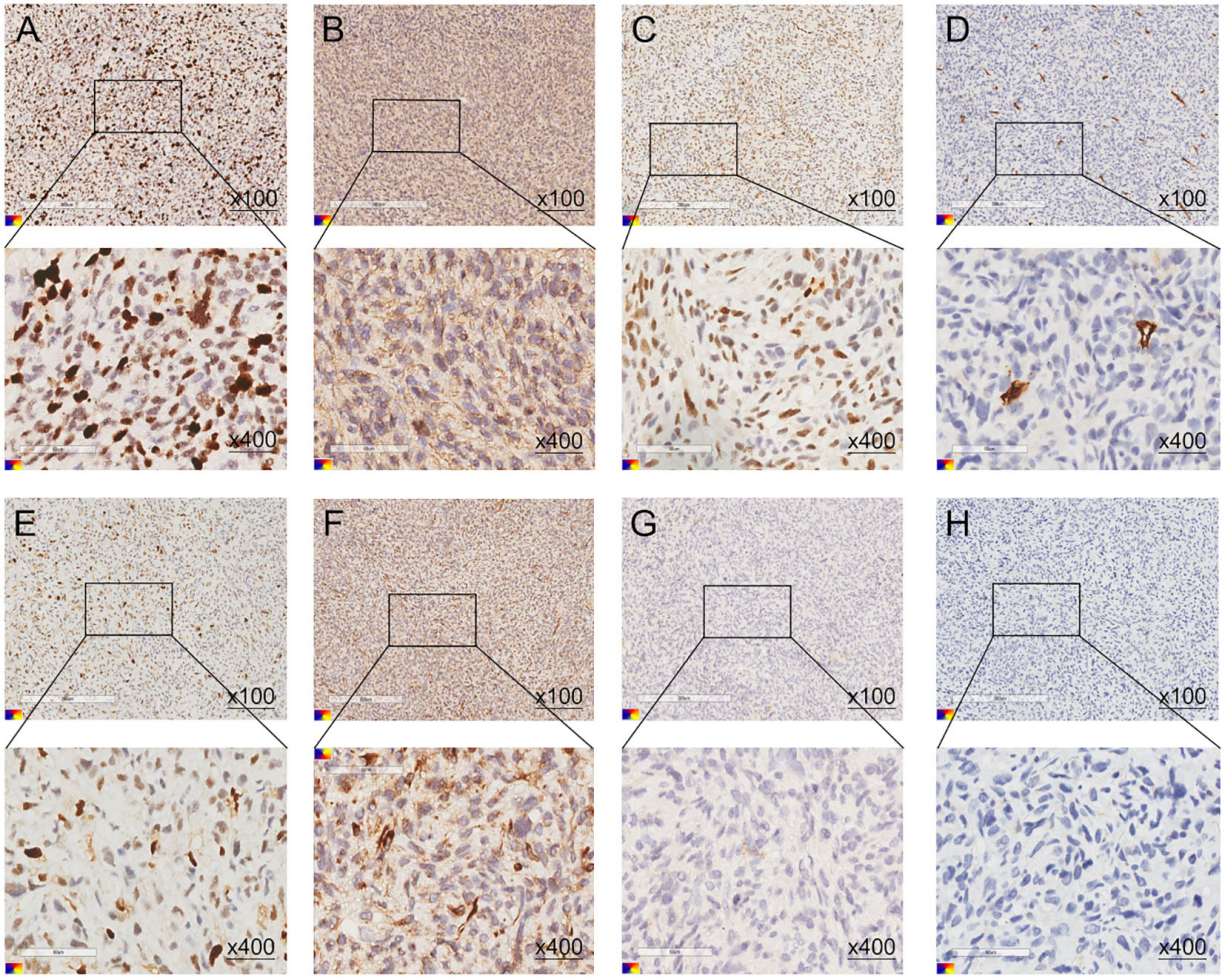
**(A–G)** The postoperative pathological immunohistochemistry results of the patient. **(A)** Ki-67 (40%, +), **(B)** SMA (++), **(C)** ERG (scattered +), **(D)** CD34 (-), **(E)** MDM2 (+), **(F)** Vimentin (++), **(G)** S100(-), **(H)** Desmin (-).

Postoperative pathological examination confirmed cardiac angiosarcoma of the left atrium, WHO Grade II classification, Immunohistochemical (IHC) staining showing positive expression for smooth muscle actin (SMA) (++), Vimentin (++), MDM2 (+), ERG (scattered +), S100(-), CD34 (-), Desmin (-), and a Ki-67 proliferation index of 40% (+) (**Figure 2**).

The patient was discharged two weeks postoperatively and returned to the Second Affiliated Hospital of Zunyi Medical University forty-three days after surgery for adjuvant therapy. Cardiac color Doppler ultrasound detected several irregular solid echogenic masses on the left atrial wall, the largest measuring approximately 40mmx14mm, also observing a 4 mm thick pericardial effusion at the posterior pericardial sac (**Figure 1. II-B**).

Following a comprehensive assessment of indications and contraindications for chemotherapy, the patient was administered the first cycle of first-line chemotherapy (doxorubicin plus cyclophosphamide) on the forty-fifth day after the surgery. Subsequently, he underwent five cycles of maintenance therapy (as before, doxorubicin in combination with cyclophosphamide) over the following six months. During this period, the patient occurred adverse events, including nausea, vomiting, and palpitation. After treatment with six cycles of chemotherapy, the largest mass on the left atrial wall decreased, measuring approximately 27mm*24mm (**Figure 1. II-C**).

In April 2024, with progressive disease (PD), the patient presented to the Second Affiliated Hospital of Zunyi Medical University for further assessment and treatment again, due to worsening symptoms of cough and shortness of breath. Echocardiography showed that three months after the completion of six cycles of chemotherapy, the mass on the left atrial wall increased from 27mm*24mm to 44mm*25mm (**Figure 1. II-D**). Second-line chemotherapy and immunotherapy were planned, but the patient refused related treatment for economic reasons. Two and a half months later, the disease progressed again. The mass increased from 44mm*25mm to 47mm*34mm (**Figure 1. II-E**). The patient refused second-line treatment for economic reasons again. Afterwards, we keep long-term follow-up after discharge, the patient remains alive up to now.

## Materials and methods

### Search strategy

Studies of potential interest were identified without language restriction by querying the electronic databases PubMed, Web of Science, Embase, and Scopus, from January 1985 to December 2023. For the database queries, three search term groups were used, with at least one term from each group required to match: (1) “heart tumor”[All Fields] OR “cardiac tumor”[All Fields] OR “heart neoplasm”[All Fields] OR “cardiac neoplasm”[All Fields]; (2) “primary angiosarcoma”[All Fields] OR “primary hemangiosarcoma”[All Fields]; (3) “postoperative”[All Fields]. No geographical restrictions were applied. We excluded studies that were abstracts, letters, reviews, commentary articles, opinion articles, studies involving pediatric populations, as well as animal and invitro studies. Finally, twenty publications were included, and all of them provided individual patient data.

### Data source

**Figure 3:**
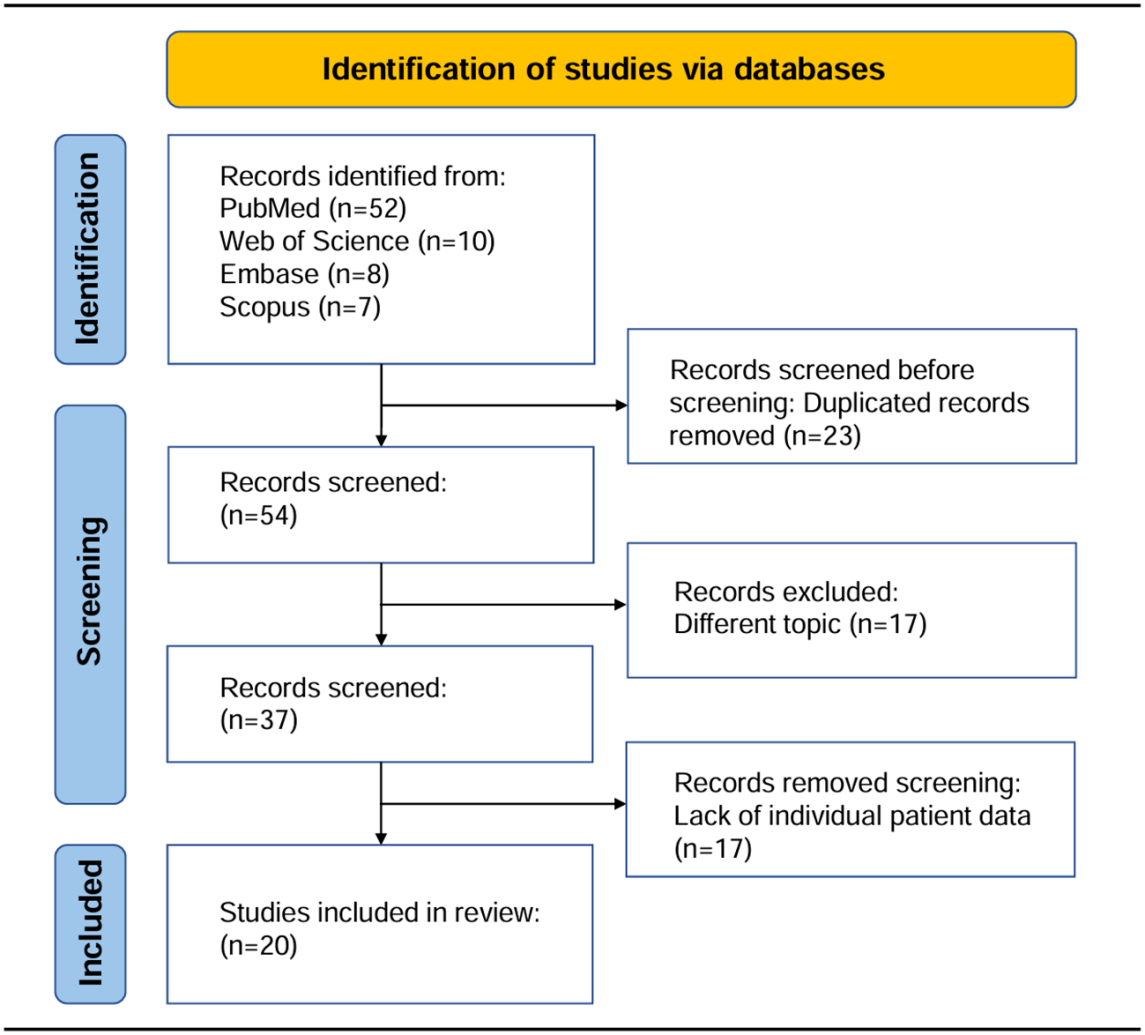
The PRISMA flow diagram illustrated the study identification and selection process for inclusion in this synthetic cohort.

We conducted a systematic review of data from 21 cases of primary cardiac angiosarcoma patients who underwent surgery, identified through comprehensive searches of PubMed, Web of Science, Embase, and Scopus databases from January 1985 to December 2023, establishing a synthetic cohort. Inclusion criteria were as follows: (1) Clear histopathological diagnosis with primary cardiac angiosarcoma; (2) no history of other cancers before the diagnosis of primary cardiac angiosarcoma; (3) all patients underwent initial surgical resection, regardless of completeness, without prior tumor-related treatment; (4) all patients had clear clinical and follow-up data. For all patients, it was clear whether they received postoperative adjuvant radiotherapy or chemotherapy. Progression-free survival (PFS) was defined as the time from initial diagnosis to disease progression or death, and overall survival (OS) was defined as the time from initial diagnosis to death or until the date of article publication. After screening, 20 publications were ultimately included in this study. Building upon this comprehensive search process, we developed a PRISMA-compliant flow chart (**Figure 3**) detailing the study identification and selection protocol for this synthetic cohort analysis. This study constitutes a systematic review methodology-based synthetic cohort analysis. The design and reporting of the research protocol for this systematic review adhere to the Preferred Reporting Items for Systematic Reviews and Meta-Analyses (PRISMA 2020) guidelines. It should be noted that this study constitutes a pooled and synthetic cohort analysis based on systematic review methodology, which differs from conventional systematic reviews. Typically, systematic reviews employ the Cochrane Risk of Bias Tool for randomized controlled trials (RCTs) and the Newcastle-Ottawa Scale (NOS) for observational cohort studies. However, as our study exclusively included case reports, neither Cochrane nor NOS tools were applicable. We therefore adopted the JBI Checklist for Case Reports for quality of included studies (2020 version, EMT Report) for quality assessment of included studies. This tool evaluated key domains including demographic characteristics, medical history and timeline, clinical presentation, diagnostic methods and results, interventions, and post-intervention outcomes. All included studies documented patient outcomes and survival status. Two investigators independently conducted the evaluations, with any discrepancies resolved through consensus discussion.

### Data analysis

Based on whether undergoing postoperative adjuvant therapy or not, the patients in the synthetic cohort were divided into two different groups. Kaplan-Meier analysis was used to evaluate the relationship between postoperative adjuvant therapy and survival in the synthetic cohort. Univariable and multivariable cox proportional hazards regression analyses were used to explore prognostic factors of primary cardiac angiosarcoma. A nomogram was constructed was constructed using the rms package (version 6.8-1) and the discrimination ability of the nomogram was evaluated by receiver operating characteristic (ROC) curves, calibration curves and decision curve analysis (DCA). The bootstrap method was used for internal validation. All statistical analyses were conducted using R software (version 4.4.1, http://www.r-project.org). P<0.05 was considered statistically significant.

### Institutional review board statement

The study was conducted in accordance with the Declaration of Helsinki. The Ethics Committees of the Second Affiliated Hospital of Zunyi Medical University approved the study protocol and publication of data (protocol code No. 2020-1-013). Patient written consent for the publication of the study data was waived by the ethical committees because the clinical record of the anonymous patients was retrospectively used. Clinical trial number: not applicable.

## Results

### Baseline characteristics

We included 21 cases of primary cardiac angiosarcoma in PubMed from January 1985 to December 2023 to establish a synthetic cohort. **Table 1** showed the baseline characteristics of these patients in postoperative management from the synthetic cohort. Patients were stratified by resection margins: 9 had R0 and 12 had R1 resections. The lesion location of most patients was right atrium (n=18, 85.71%). A total of 9 patients received radiotherapy (42.86%) and ten patients received chemotherapy (47.62%). More than a half of the patients developed distant organ metastasis (n=11, 52.38%).

**Table 1:** Baseline characteristics of 21 patients in postoperative management from the synthetic cohort.

Author	Age	Sex	Treatment	Follow-up(m)	Lesion location	Survival status	Nation	Immunohistochemistry	CT	RT (Gy)	Metastasis
Park, K. S.	47	M	R0, RT, CT	24	right atrium	Alive	Japan	CD31(+++)	Unknown	Unknown	No
Linfeng, Q.	65	M	R1	10	right atrium	Died	China	CD34(+), CD31(+), F8-R-Ag(+),Vimentin(+)	No	No	brain
Bakr, L.	22	M	R1, RT, CT	12	right atrium	Died	Syria	Unknown	Unknown	Unknown	brain,lung
Moeri-Schimmel, R.	20	M	R0, RT, CT	12	right atrium	Alive	Netherlands	Unknown	paclitaxel 80 mg/m2 days 1–8–15 q28 days	33×1.8 Gy	lung
Bellitti, R.	25	M	R1, RT, CT	18	right atrium	Alive	Italy	vimentin(+), CD31(+), CD34(+), actin(+),focal factor VIII(+),c-kit(+),EGFR(-)	4 cycles of chemotherapy with gemcitabine and docetax, c-kit inhibitor (imatinib) for 3 months	Unknown	No
Brandt, R. R.	63	F	R0, CT	10	right atrium	Died	Germany	CD31(+), CD34(+), Vimentin(+)	4 cycles of AIG (adriamycin [doxorubicin], ifosfamide, and granulocyte colony stimulating factor) combination chemotherapy followed by second-line treatment with paclitaxel	No	Unknown
Yada, M.	52	M	R1, RT, CT	24	right atrium	Alive	Japan	CD31(+), CD34(+), D2-40(+), ERG transcription factor(+)	weekly paclitaxel treatment 80 mg/m2)	30×2.0 Gy	liver
Do, T. H.	44	M	R0, CT	6	left atrium	Alive	America	CD34(+), CD31(+), ERG(+)	Unknown	No	No
Uchita, S.	66	M	R1	8	right atrium	Died	Japan	Unknown	No	No	Unknown
Blindaru, A.	18	F	R1, RT, CT	9	right atrium	Alive	America	CD31(+), CD34(+), ERG(+), Ki67(+)	ifosfamide (3,000 mg),mesna (2,800 mg),doxorubicin (40 mg), 6 cycles	Unknown	brain,lung
Shimada, N.	34	F	R1, CT	14	right atrium	Died	Japan	Unknown	Doxorubicin hydrochloride/ifosfamide	No	brain,lung
Poonia, A.	62	F	R0	6	left atrium	Died	India	Unknown	No	No	No
Kugai, T.	56	M	R0, RT, CT	14	right atrium	Died	Japan	Unknown	Unknown	Unknown	Unknown
Hattori, Y.	38	M	R1	1	right atrium	Died	Japan	vimentin(+), EMA(-), keratin(-)	No	No	No
Slepicka, C.	35	F	R1, RT	8	right atrium	Alive	Africa	Unknown	No	Unknown	bone,peritoneum
Kuwabara, F.	61	F	R1	6	left atrium	Died	Japan	Unknown	No	No	Adrenal gland
Crespo, M. G.	31	M	R0	8	right atrium	Died	Spain	Unknown	No	No	brain
Crespo, M. G	32	M	R0	9	right atrium	Died	Spain	Unknown	No	No	brain
Velloso, L. G.	19	F	R0, RT	5	right atrium	Died	Portugal	Unknown	No	Unknown	Unknown
Grollier, G.	70	F	R1	9	right atrium	Died	France	Unknown	No	No	brain,liver
Kawahito, K.	57	M	R1	5	right atrium	Died	Japan	Unknown	No	No	No

F, female; M, male; R indicated the resection status in the surgery, R0, R0 resection; R1, R1 resection; RT, radiotherapy; CT, chemotherapy (involving a combination of doxorubicin and ifosfamide); RT and CT, radiotherapy in combination with chemotherapy. The relevant literature mentioned in **Table 1** listed in reference (9–28).

### Survival analysis for the patients in the synthetic cohort

**Figure 4:**
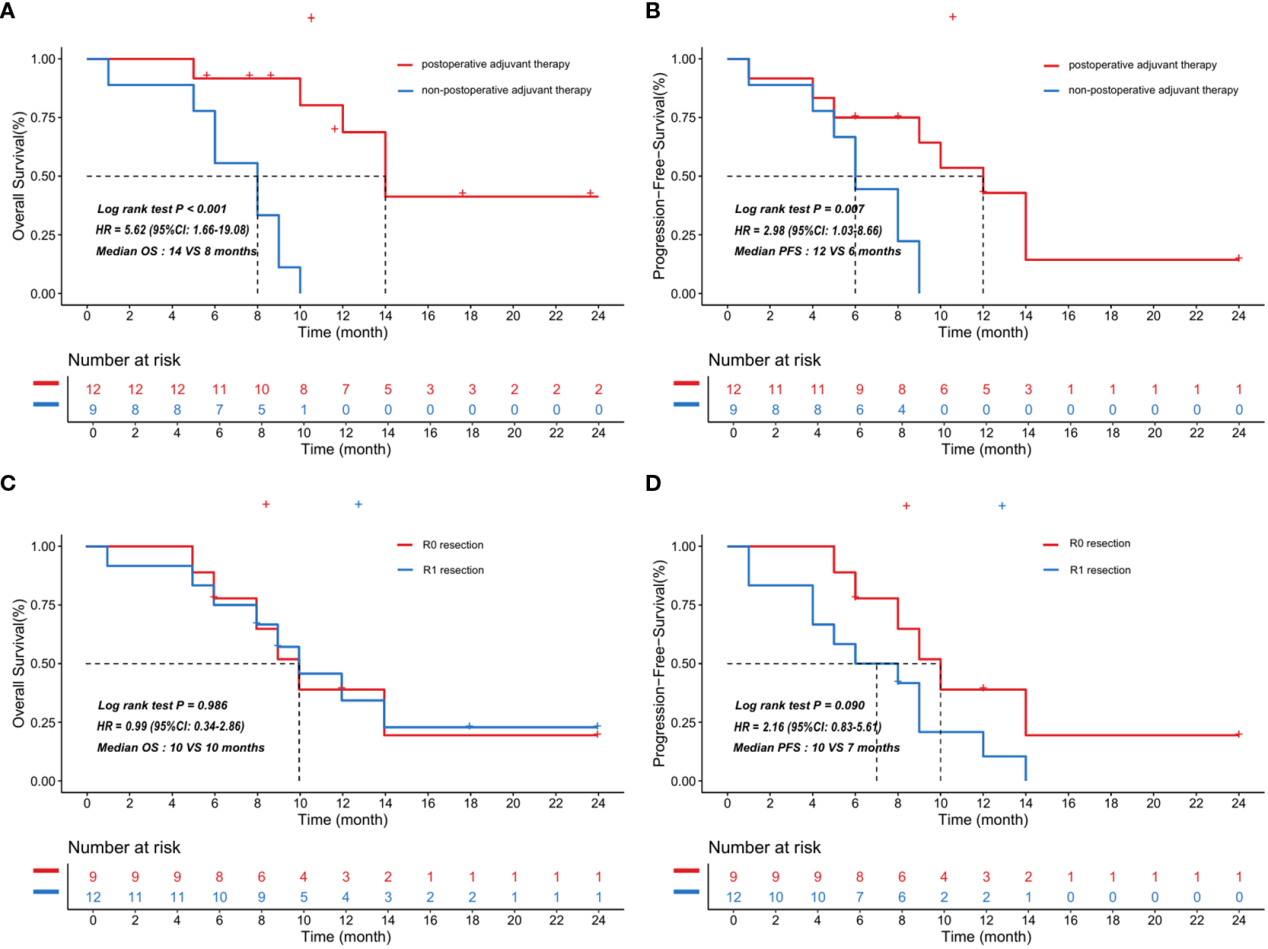
Kaplan-Meier curves showed the correlation between postoperative adjuvant therapy and survival in the synthetic cohort with 21 patients. The patients treated with postoperative adjuvant therapy showed significantly better overall survival **(A)** and progression-free survival **(B)** compared with the patients who did not. The curves also indicated that overall survival **(C)** was indistinguishable between patients with R0 or R1 resection in the synthetic cohort, but patients underwent R0 resection had longer but not statistically significant progression-free survival **(D)** compared to patients underwent R1 resection. HR and p value obtained by log rank test, HR: hazard ratio, 95% CI: 95% confidence interval, OS: overall survival, PFS: progression-free survival.

We next explored the association between postoperative adjuvant therapy, tumor complete resection and prognosis of patients with primary cardiac angiosarcoma. We divided 21 patients in the synthetic cohort into two groups based on whether they received postoperative adjuvant therapy. Similarly, the 21 patients were categorized into two groups based on whether they had undergone complete tumor resection. In **Figure 4**, survival analysis indicated that patients with postoperative adjuvant therapy showed significantly better survival compared with patients without postoperative adjuvant therapy, in terms of both overall survival (median OS, 14 VS 8 months, HR = 5.62; 95%CI: 1.66-19.08; P<0.001) and progression-free survival (median PFS, 12 VS 6 months, HR = 2.98; 95%CI: 1.03-8.66; P = 0.007). Meanwhile, the results revealed that OS was indistinguishable between patients with R0 or R1 resection in surgery (median OS, 10 VS 10 months, HR = 0.99; 95%CI: 0.34-2.86; P = 0.986). However, patients underwent R0 resection had longer but not statistically significant PFS compared to patients underwent R1 resection (median PFS, 10 VS 7 months, HR = 2.16; 95%CI: 0.83-5.61; P = 0.090).

### Radiotherapy and chemotherapy were prognostic risk factors in primary cardiac angiosarcoma

Univariable and multivariable cox regression analyses were carried out with 21 patients from the synthetic cohort. Univariable analysis indicated radiotherapy (HR = 0.14; 95% CI: 0.04-0.54; P = 0.004) and chemotherapy (HR = 0.03; 95% CI: 0.00-0.27; P = 0.002, **Table 2**.) were prognostic factors of OS. Multivariable analysis showed chemotherapy was an independent risk factor for patient prognosis (HR = 0.03; 95% CI: 0.00-0.34; P = 0.005), while radiotherapy was not (HR, 0.43; 95% CI: 0.09-2.09; P = 0.293, **Supplementary Table S1**). Age, sex, resection, location and metastasis were not associated with survival in patients with primary cardiac angiosarcoma (P>0.05.).

**Table 2:** Univariable cox analysis of OS in primary cardiac angiosarcoma patients from the synthetic cohort.

Features	Univariable analysis		
	HR	95% CI	p value
Age (≤50 vs. >50)	1.96	0.68-5.68	0.214
Sex (Male vs. Female)	0.54	0.18-1.61	0.271
Resection (R0 vs. R1)	1.03	0.36-2.96	0.964
Location (Left vs. Right atrium)	0.26	0.04-1.55	0.138
Chemotherapy (Yes vs. No)	0.03	0.00-0.27	0.002
Radiotherapy (Yes vs. No)	0.14	0.04-0.54	0.004
Metastasis (Yes vs. No)	1.40	0.62-3.15	0.416

### Nomogram based on radiotherapy and chemotherapy for prognostic prediction of primary cardiac angiosarcoma

**Figure 5:**
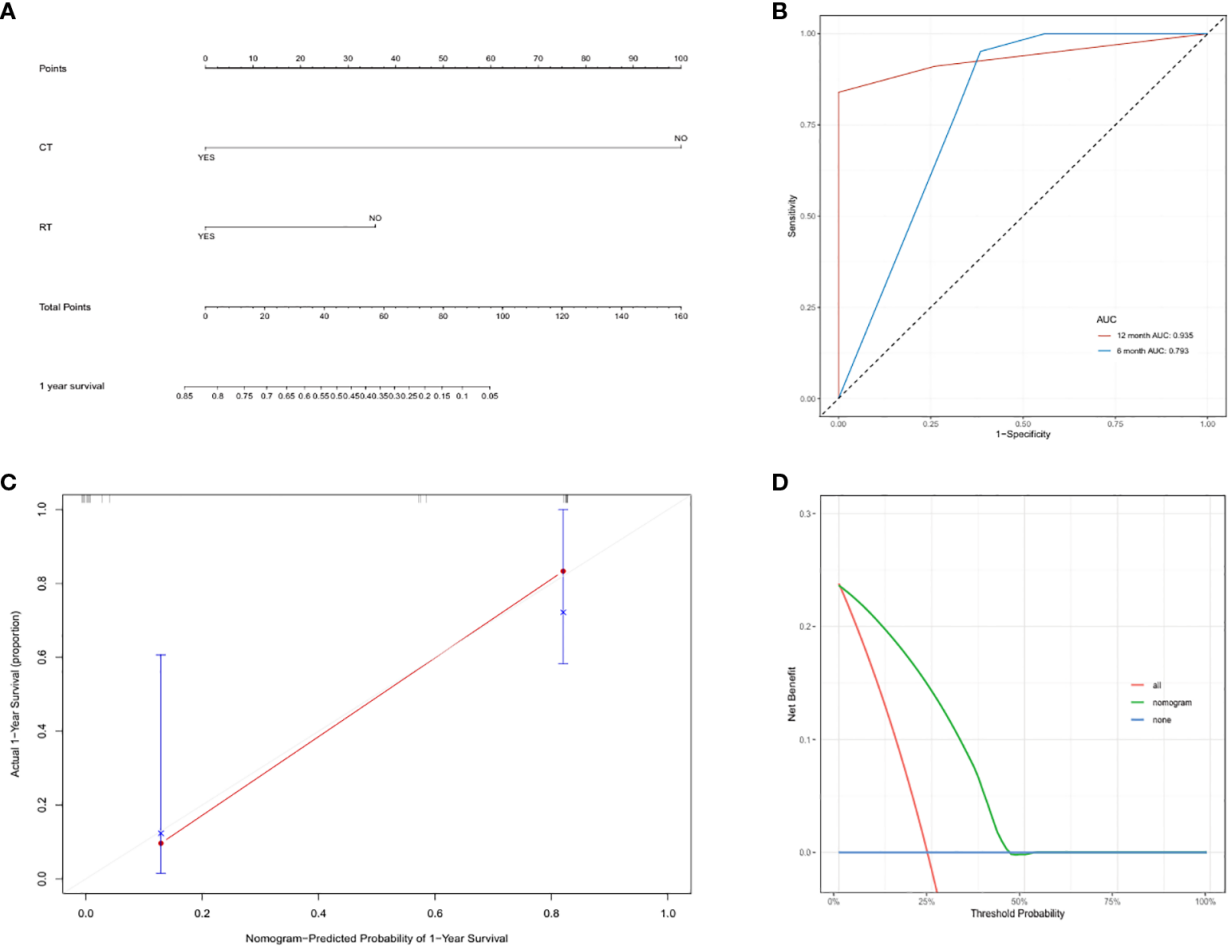
**(A)** Nomogram to predict overall survival in primary cardiac angiosarcoma patients: based on radiotherapy and chemotherapy for 1-year overall survival prediction. **(B)** Time-dependent ROC curves of the nomogram. We used AUC values at 6-, 12-month to assess prognostic accuracy using the log-rank test. ROC: receiver operator characteristic. AUC: area under the ROC curve. **(C)** The calibration curve of the nomogram for predicting 1-year overall survival in the synthetic cohort. Nomogram-predicted OS is plotted on the x-axis; actual OS is plotted on the y-axis. OS: overall survival. **(D)** DCA curve of the nomogram for postoperative 6-month survival prediction of primary cardiac angiosarcoma. DCA: Decision curve analysis.

On the basis of the results of the univariable and multivariable analysis, we selected variables with statistical significance. A nomogram including radiotherapy and chemotherapy to predict the survival of patients with primary cardiac angiosarcoma was constructed (**Figure 5A**). The bootstrap method was used to validate the model internally. The time-dependent AUC of 6-, 12-month survival for the nomogram were 0.793 and 0.935, respectively (**Figure 5B**). Calibration of the nomogram for 12-month survival was performed by comparing the predicted survival with the observed survival after bias correction. The calibration curves of the nomogram illustrated consistency between the observed and predicted results, indicating that the predicted 12-month survival of patients with postoperative management of primary cardiac angiosarcoma is generally consistent with the actual risk, albeit with a slight overestimation (O/E index: 0.65, **Figure 5C**). The DCA curve showed that if the threshold probability was between 3% and 42%, the nomogram model would provide a net benefit (threshold probability 0.03, net benefit 0.229; threshold probability 0.42, net benefit 0.031, **Figure 5D**). The ROC, calibration and DCA curves suggested that the prediction model had high accuracy and good discrimination.

## Discussion

### Diagnosis in cardiac angiosarcoma

Primary cardiac angiosarcoma is extremely rare, with an estimated incidence of approximately 0.034 per 100,000 in the population (22). Cardiac sarcoma primarily occurs in individuals aged 30-50, with a significantly higher incidence in males than females and a male-to-female ratio of approximately 3:1 (4). Clinical presentations of cardiac malignancies are typically nonspecific and often correlate with tumor size and location. Common symptoms include heart failure due to blood flow obstruction or dyspnea and cough associated with pulmonary artery hypertension. Most patients are initially admitted for respiratory-related issues, and further investigation reveals the presence of malignant cardiac tumors. Some patients may exhibit a range of systemic symptoms due to ischemia or pericardial invasion (29, 30). While echocardiography is the most cost-effective and sensitive diagnostic tool for cardiac malignancies, magnetic resonance imaging and computed tomography scans are indicated when a comprehensive assessment of tumor extension and adjacent structures is required (31).

### Treatment and prognosis management in cardiac angiosarcoma

There are no consensus or guidelines about primary cardiac angiosarcoma management. Currently, surgery is generally considered the most effective treatment. However, primary cardiac angiosarcoma still poses therapeutic challenges to cardiac surgeons and oncologists because of the technical difficulties involved in extensive cardiac resections and the aggressive biological nature of the tumor. Patients who underwent complete surgical resection had a median survival of approximately 17 months, while those with incomplete resection had a median survival of only 6 months (32). Local recurrence has been low with complete resection, but distant metastasis remains the prominent cause of death (33). In this study, we noted that patients underwent R0 resection presented with a longer but not statistically significant PFS, cluing that tumor uncomplete resection would affect the disease progression, which was consistent with recent study by Cho et al. showing the beneficial effects of complete resection on patient survival (34). Meanwhile, our study showed that patients with postoperative adjuvant therapy had a significantly better survival compared with patients without adjuvant therapy. Several studies have reported that postoperative adjuvant therapy significantly prolonged survival in patients with primary cardiac sarcoma (8, 34, 35). Stergioula et al. (36) analyzed the outcome of 127 studies containing data for 162 patients over a 34-year period and showed that multimodality treatment that incorporated surgery and adjuvant chemo-radiotherapy was associated with a statistically significant survival benefit. Notably, the study also observed longer median PFS and OS in patients who underwent macroscopically complete resection, although statistical significance was not reached. This finding aligned with our results regarding the potential benefits of R0 resection. Despite differences in resection margin subgroup classifications between the studies, the consistent trend further supported the value of complete resection from complementary perspectives. Previous research has confirmed that adjuvant therapy combining ifosfamide and doxorubicin, or doxorubicin with cisplatin, provides substantial benefits for patients with metastatic or unresectable angiosarcoma (37). The combination of surgery and neoadjuvant chemotherapy for cardiac sarcomas achieved a median survival of 20 months, while that of patients without neoadjuvant chemotherapy is only 9.5 months (38). These results were consistent with our study.

Our study revealed a significant prolongation of OS and PFS in patients treated with postoperative adjuvant therapy, including chemotherapy, radiotherapy, regardless of whether patients underwent R0 resection. Meanwhile, the patient we reported remains alive with tumor after undergoing surgery and six cycles of regular chemotherapy, from May 2023 to now. The above results clued that postoperative adjuvant therapy would benefit to patients with primary cardiac angiosarcoma. Furthermore, our study suggested chemotherapy was an independent risk factor for patient prognosis, which was consistent with prior research results (39). After treatment with six cycles of chemotherapy, the mass on the left atrial wall of the patient we reported decreased, confirming the benefit of chemotherapy for patients with primary cardiac angiosarcoma. Previous research reported that being ≥ 45 years, and having > 5 *cm* tumors were associated with lower survival. In our study, we found no significant correlation between age, sex, location, metastasis, and survival, which contrasted with Zhang et al.’s claim regarding the correlation between age and OS (40). The ROC, calibration and DCA curves showed that the prediction model based on radiotherapy and chemotherapy had high accuracy and discrimination, which would improve the current situation of risk assessment and benefit the individualized clinical decision.

### Biomolecular targets in cardiac angiosarcoma

Primary cardiac angiosarcoma is a rare subtype of soft-tissue sarcomas characterized by dismal prognosis. VEGF and its receptors were the most studied targets in angiosarcoma. Originating from vascular endothelial cells, there is significant interest in the role of angiogenesis and associated angiogenic factors in angiosarcoma pathogenesis, as well as in how they might be used as targets for treatment. Wagner et al. reported that MAPK signaling was found active in over half of clinical angiosarcoma samples, and combined inhibition of the VEGF and MAPK pathways with cediranib and trametinib had an additive effect in angiosarcoma (41). Wang et al. demonstrated that the combination of anlotinib and epirubicin significantly inhibited tumor growth in the advanced soft-tissue sarcoma (42, 43). Studies by Palassini et al. reported that Gemcitabine-based chemotherapy had a significant response on OS of patients with localized radiation-associated angiosarcoma of the breast region (44).

No comprehensive studies of molecular changes in angiosarcoma have been published. Gene-expression microarray technology could help to identify unique molecular signatures for histological subtypes, and improve the understanding of key molecular events in the pathogenesis of soft-tissue tumors, and suggest potential therapeutic targets (45). Khor et al. found that secreted phosphoprotein 1 (SPP1) overexpression would be a potential biomarker of chemoresistance and poor prognosis in angiosarcoma (46). Focusing on the rare and aggressive cardiac sarcomas, our team identified several novel clinical markers by spatial transcriptomics, such as immunoglobulin kappa C (IGKC), procollagen C-endopeptidase enhancer (PCOLCE), Neuroepithelial transforming gene 1 (NET1), transducing-like enhancer protein 2 (TLE2), troponin C1 (TNNC1), and calponin 3 (CNN3), that classify normal heart tissue from sarcoma subtypes (47). Charles et al. identified ERG as the most sensitive marker for patients with cardiac angiosarcoma through comprehensive genomic analysis of ten patients, and revealed trisomy involving chromosomes 8, 20, CDKN2 homozygous deletion, and 1q in patients with cardiac angiosarcoma (48). Trisomy in chromosome 20 or 8 was associated with the promotion of tumor survival by regulating PLCgamma 1 (PLCG1) and MYC (49, 50). In neuroblastoma and multiple myeloma, the presence of 1q+ often indicated an unfavorable prognosis (51, 52). However, in patients with gliomas, those with 1q+ showed increased sensitivity to chemotherapy (53). In cardiac angiosarcoma, three patients with 1q+ had better survival of 13.4 to 61.2 months after chemotherapy, while non-chemotherapy patients with 1q+ survived only 3.16 months (48). The above results suggested that chemotherapy would be beneficial to patients with 1q+ cardiac angiosarcoma. Trisomy in chromosomes 8, 20, and 1q+ to some extent can reflect the prognosis, but the specific mechanisms require further research. Huo et al. performed scRNA-seq analysis and revealed significant intratumoral heterogeneity in primary cardiac angiosarcoma driven by diverse biological processes such as protein synthesis, degradation, and Retinoic acid-inducible gene I (RIG-I) signaling inhibition (54). The Angiosarcoma Project will promote the in-depth research of this tumor, which would generate and publicly release clinically annotated genomic data on tumor and germline specimens on an ongoing basis (55).

### Limitations and future directions

Our study has inherent limitations. The model was only subjected to bootstrap internal validation and O/E index should be interpreted with caution, due to the small limited sample size of rare cardiac tumor patients. The sample size was small due to the low incidence and may not have afforded sufficient statistical efficacy. The survival estimates at the tail of the curves may be less reliable due to the small number of patients at risk. Although the results of the performed analysis should be interpreted with caution due to the statistical heterogeneity of the used patient cohort, this study contributes to multi-center, large-sample research in the future by presenting the findings and trends identified in a small sample size. We discussed the relevance between tumor complete resection, postoperative adjuvant therapy and survival. The included studies consistently reported key domains including demographic characteristics, medical history, clinical presentation, diagnostic methods and results, interventions, and post-intervention outcomes. All studies explicitly documented patient outcomes and survival status. However, certain studies lacked comprehensive clinical characterization, particularly regarding tumor size and stage details, thus lacking related data analysis. In fact, tumor size, location and stage were also crucial factors influenced prognosis of patients. Studies by Liu, Zhang, and Kamitani et al. have confirmed that tumor size, stage and comprehensive treatment were independently prognostic factors for primary angiosarcoma (15, 56, 57). Additionally, due to the lack of consensus on the management of primary cardiac angiosarcoma, patients were treated with variable treatment protocols, including different surgical resection and chemo-radiotherapy regimens. It is possible that patients who received adjuvant therapy had a better prognosis to start with compared to those who were not eligible and thus not considered for radiotherapy and chemotherapy. The included studies cover an extended period (1985–2023), during which individual chemotherapy protocols varied with evolving understanding of cardiac sarcomas and influenced patient survival. Future research should further investigate which chemotherapy protocol contributes most to postoperative survival. The rarity and heterogeneity of primary cardiac angiosarcoma pose formidable challenges in identification for effective biomarkers and treatment, resulting in limited progress in survival rates in recent decades (58). Advanced imaging techniques, biomarkers, and immunohistochemical analysis assist in confirming the diagnosis and guiding treatment decisions (59). We strongly recommend performing multi-institutional prospective cohorts to better study disease course and treatments to develop consensus, algorithms, and guidelines for this type of sarcoma. With the rapid advancement of oncogenomic, it would provide more guidance for the treatment and management of primary cardiac angiosarcoma.

## Conclusions

The prognosis of patients with primary cardiac angiosarcoma remains extremely poor, despite surgical intervention. Postoperative adjuvant therapy was associated with significantly better survival in a small cohort of patients with primary cardiac angiosarcoma. Further studies are warranted to guide future recommendations.

## Data Availability

The original contributions presented in the study are included in the article/**Supplementary Material**. Further inquiries can be directed to the corresponding author.
