# Supplementary material for: A synthetic cohort analysis of postoperative management of primary cardiac angiosarcoma and a case report
Source: Front Oncol. 2025 Sep 15;15:1625049. doi: 10.3389/fonc.2025.1625049 (PMC12478302; doi:10.3389/fonc.2025.1625049)
Supplement: Supplementary file 1 [file Table1.docx]

A synthetic cohort analysis of postoperative management of primary cardiac angiosarcoma and a case report

Ying Cai ^1,†^, Hang Yang ^1,†^, Dan Yuan ^2^, Su-Han Jin ^3^, Junzhu Xu ^1^, Wei Hu ^1^, Yuju Bai ^1^, Xinjuan Li ^4^, Zejin Wang ^5^, Dengshen Zhang ^6^, Ke Guo ^6^, Shixiang Wang ^7^, Udo S Gaipl ^8^, Yuan Liu ^9, ‡^, Hu Ma ^1, ‡^, Jian-Guo Zhou ^1,8, ‡,*^

1. Department of Oncology, The Second Affiliated Hospital of Zunyi Medical University, Zunyi, 563000, P. R. China; Ying Cai, [cy19980404@aliyun.com](mailto:cy19980404@aliyun.com); Hang Yang, [zmuyanghang@126.com](mailto:zmuyanghang@126.com); Wei Hu, [huwei@zmu.edu.cn](mailto:huwei@zmu.edu.cn); Yuju Bai, [byj6618@163.com](mailto:byj6618@163.com); [Junzhu Xu](https://pubmed.ncbi.nlm.nih.gov/?term=Xu+J&cauthor_id=38304028), [xujunzhu959@163.com](mailto:xujunzhu959@163.com); Hu Ma, [mahuab@163.com](mailto:mahuab@163.com); Jian-Guo Zhou, [jianguo.zhou@zmu.edu.cn](mailto:jianguo.zhou@zmu.edu.cn).
2. Department of Pathology, Affiliated Hospital of Zunyi Medical University, Zunyi, 563000, P. R. China; Dan Yuan, [284234442@qq.com](mailto:284234442@qq.com).
3. Department of Orthodontics, Affiliated Stomatological Hospital of Zunyi Medical University, Zunyi, P. R. China; Su-Han Jin, [doctorjin1991@163.com](mailto:doctorjin1991@163.com).
4. Department of Imaging, The Second Affiliated Hospital of Zunyi Medical University, Zunyi, 563000, P. R. China; Xinjuan Li, [18798120251@163.com](mailto:18798120251@163.com).
5. Department of Ultrasound, The Second Affiliated Hospital of Zunyi Medical University, Zunyi, 563000, P. R. China; Zejin Wang, [2573781250@qq.com](mailto:2573781250@qq.com).
6. Department of Cardiac Macrovascular Surgery, Affiliated Hospital of Zunyi Medical University, Zunyi, 563000, P. R. China. Ke Guo, [273434952@qq.com](mailto:273434952@qq.com). Dengshen Zhang, [xwkzds2021@163.com](mailto:xwkzds2021@163.com).
7. Department of Biomedical Informatics, School of Life Sciences, Central South University, Changsha, PR China. Shixiang Wang, [wangshx@csu.edu.cn](mailto:wangshx@csu.edu.cn).
8. Translational Radiobiology, Department of Radiation Oncology, Universitätsklinikum Erlangen, Friedrich-Alexander-Universität Erlan-gen-Nürnberg, Erlangen, Germany; Udo S Gaipl, [udo.gaipl@uk-erlangen.de](mailto:udo.gaipl@uk-erlangen.de).
9. Department of Clinical Pharmacy, Liaocheng Cancer Hospital, Liaocheng 252000, P. R. China; Yuan Liu, [zmc_liuy@163.com](mailto:zmc_liuy@163.com).

† These authors contributed equally to this work as first authors.

‡ These authors contributed equally to this work as last authors.

* Correspondence: Jian-Guo Zhou, [jianguo.zhou@zmu.edu.cn](mailto:jianguo.zhou@zmu.edu.cn).

Supplementary Tables

[**Supp Table S1** 3](#_Toc129037092)

[**Supp Table S2** 4](#_Toc129037093)

**Supplementary Table S1.** Multivariable cox analysis of overall survival in primary cardiac angiosarcoma patients from the synthetic cohort

| **Features** | **Multivariable analysis** | | |
| --- | --- | --- | --- |
|  | **HR** | **95% CI** | **p value** |
| **Age (≤50 vs. ＞50)** | 0.71 | 0.16-3.12 | 0.648 |
| **Sex (Male vs. Female)** | 0.55 | 0.13-2.28 | 0.412 |
| **Resection (R0 vs. R1)** | 1.80 | 0.47-6.95 | 0.394 |
| **Location (Left vs. Right** **atrium)** | 0.32 | 0.02-4.25 | 0.385 |
| **Chemotherapy (Yes vs. No)** | 0.03 | 0.00-0.34 | 0.005 |
| **Radiotherapy (Yes vs. No)** | 0.43 | 0.09-2.09 | 0.293 |
| **Metastasis (Yes vs. No)** | 2.18 | 0.62-7.71 | 0.226 |

**Supplementary Table S2. Treatment timeline for the patient**

| June 14th, 2023 | A 49-year-old man was admitted to the local hospital for cough, sputum production, exertional dyspnea, and bilateral lower extremity edema after exposure to cold in the past three weeks. After two weeks of anti-infective therapy (levofloxacin, cephalosporins, and symptomatic treatment with antitussive medications), the symptoms showed no improvement. Then the patients presented to the Respiratory Department of the Affiliated Hospital of Zunyi Medical University for further treatment. Upon evaluation, the physical examination revealed a normal cardiac silhouette, a heart rate of 78 bpm with regular rhythm, and no audible cardiac murmurs on auscultation. Chest computed tomography (CT) was performed to detect pulmonary infection, leading to anti-infective therapy in the department. |
| --- | --- |
| June 16th, 2023 | After anti-infective therapy, the patient's pulmonary infection improved. Further examination with Cardiac color Doppler echocardiography revealed the following findings:  Left Atrial Mass: A hypoechoic mass of approximately 30mmx23mm was detected in the left atrium, attached to the base of left atrial appendage, exhibiting dynamic activity synchronized with the cardiac cycle and obstructing the diastolic mitral valve.  Pericardial Effusion: Hypoechoic areas measuring approximately 4mm and 5mm in thickness were detected in the anterior pericardium and below the cardiac apex, respectively.  Mitral Valve Stenosis: The echocardiogram revealed mitral valve obstructive stenosis with a forward flow velocity of approximately 311 cm/s and a peak pressure gradient of about 39 mmHg. Mild tricuspid regurgitation was present, and the estimated pulmonary artery systolic pressure, based on tricuspid regurgitation, was approximately 61 mmHg.  These findings suggested significant cardiac lesions, including a mass in the left atrium, pericardial effusion, and mitral valve stenosis, emphasizing the necessity for further assessment and management. |
| June 16th, 2023 | The patient was transferred to the Department of Cardiovascular Surgery at the Affiliated Hospital of Zunyi Medical University for further treatment. |
| June 25th, 2023 | After a thorough evaluation and exclusion of surgical contraindications, the patient underwent open heart surgery under general anesthesia. Postoperative pathological examination indicated cardiac angiosarcoma of the left atrium, WHO Grade II classification. IHC revealed positive expression for Vimentin (++), smooth muscle actin (SMA) (++), Vimentin (++), MDM2 (+), ERG (scattered +), S100(-), CD34 (-), Desmin (-), and a Ki-67 proliferation index of 40% (+). |
| July 11th, 2023 | Discharged from the Department of Cardiovascular Surgery at the Affiliated Hospital of Zunyi Medical University. |
| August 8th, 2023 | The patient presented at the Second Affiliated Hospital of Zunyi Medical University for further postoperative adjuvant therapy. The echocardiogram revealed multiple irregular solid echo masses on the left atrial wall, the largest measuring approximately 40mm×14mm, and approximately 4-mm-thick pericardial effusion at the posterior pericardium. |
| August 10th, 2023 | The patient was administered the first cycle of ﬁrst-line chemotherapy (doxorubicin in combination with cyclophosphamide). |
| September 6th, 2023 | Perform the second cycle of chemotherapy (as before, doxorubicin in combination with cyclophosphamide). |
| October 17th, 2023 | Perform the third cycle chemotherapy (as before, doxorubicin in combination with cyclophosphamide). |
| November 15th, 2023 | Perform the fourth cycle chemotherapy (as before, doxorubicin in combination with cyclophosphamide). |
| December 13th, 2023 | Perform the fifth cycle chemotherapy (as before, doxorubicin in combination with cyclophosphamide). |
| January 9th, 2024 | Perform the sixth cycle chemotherapy (as before, doxorubicin in combination with cyclophosphamide). |
| January 12th, 2024 to April 7th, 2024 | The patient was discharged. After treatment with six cycles of chemotherapy, the largest mass on the left atrial wall decreased, measuring approximately 27mm*24mm. Then we keep long-term follow-up after discharge. |
| April 7th, 2024 | The patient presented to the Second Affiliated Hospital of Zunyi Medical University for further assessment and treatment again, due to worsening symptoms of cough and shortness of breath. Echocardiography showed the mass on the left atrial wall increased, from 27mm×24mm to about 44mm×25mm, compared to January 12th, 2024. Second-line chemotherapy and immunotherapy were planned, but the patient refused related treatment for economic reasons. |
| June 24th, 2024 | The patient presented at the Second Affiliated Hospital of Zunyi Medical University again, due to worsening symptoms of cough and shortness of breath. Echocardiography showed the mass on the left atrial wall increased again, from 44mm×25mm to about 47mm×34mm, compared to April 7th, 2024. Cardiac magnetic resonance imaging (MRI) showed a round mass in the left atrium, unenhanced on contrast, with bilateral pleural effusions. Second-line chemotherapy and immunotherapy were planned, but the patient refused for economic reasons again. |
| Up to now | The patient is still alive with tumor. We keep long-term follow-up after discharge. |
